# Clustering analysis of hamstring injuries—A pilot study to determine phenogroups based on injury characteristics and recovery duration

**DOI:** 10.1002/jeo2.70885

**Published:** 2026-08-17

**Authors:** Bálint Zsidai, György Gulácsi, Tibor Rudisch, Bence Márk Kovács, Pete Friar, Richard Nagy, Robert de Jonge, Eric Hamrin Senorski, Kristian Samuelsson, Gergely Pánics

**Affiliations:** ^1^ Department of Orthopaedics, Institute of Clinical Sciences, Sahlgrenska Academy University of Gothenburg Gothenburg Sweden; ^2^ Department of Orthopedics Skåne University Hospital Malmö/Lund Sweden; ^3^ Division of Musculoskeletal Imaging, Medical Imaging Center Semmelweis University Budapest Hungary; ^4^ South‐Pest Hospital Centre Budapest Hungary; ^5^ Ferencvárosi TC Health Performance & Research Hub Budapest Hungary; ^6^ Budapesti Uzsoki Street Hospital Budapest Hungary; ^7^ Department of Traumatology Semmelweis University Budapest Hungary; ^8^ FIFA Medical Centre of Excellence Budapest Hungary; ^9^ Unit of Physiotherapy, Department of Health and Rehabilitation, Institute of Neuroscience and Physiology, Sahlgrenska Academy University of Gothenburg Gothenburg Sweden; ^10^ Sportrehab Sports Medicine Clinic Gothenburg Sweden; ^11^ Department of Orthopaedics Sahlgrenska University Hospital Mölndal Sweden

**Keywords:** cluster analysis, hamstring injuries, machine learning, magnetic resonance imaging, return to play

## Abstract

**Purpose:**

The objective of this pilot study was to determine clinically relevant phenogroups of professional football players with hamstring injuries using unsupervised cluster analysis based on demographic and injury‐related characteristics, and to assess return to play (RTP) duration with respect to the characteristics of these phenogroups.

**Methods:**

This retrospective cohort study analysed 57 professional football players from Ferencvárosi Torna Club with magnetic resonance imaging (MRI)‐confirmed acute hamstring injuries reported between 2018 and 2024. Three clustering approaches were implemented and compared: k‐means clustering, hierarchical clustering using Ward's linkage method, and mixed‐type clustering using Gower distance with partitioning around medoids (PAMs). The optimal number of clusters was determined using silhouette analysis. Between‐cluster differences in RTP duration and injury characteristics were assessed using Kruskal–Wallis tests for continuous variables and chi‐square tests for categorical variables, with post‐hoc Bonferroni correction.

**Results:**

Mixed‐type clustering identified seven phenogroups based on British Athletics Muscle Injury Classification (BAMIC) grade, lesion length, anatomical tear site, affected muscle and injury extensiveness. The average silhouette width was 0.290, indicating weak to moderate cluster separation. Clusters 1, 3, 4, 5 and 6 were characterised by low‐ to moderate‐grade injury parameters and median RTP durations ranging from 9 to 27 days. Clusters 2 and 7 displayed high‐grade injury characteristics with considerably prolonged RTP (medians of 46.5 and 94.5 days, respectively). Significant between‐cluster differences were observed for days to RTP, lesion length, BAMIC grade, injury severity and anatomical tear site (*p* < 0.05).

**Conclusions:**

Unsupervised machine learning may help identify clinically relevant hamstring injury phenogroups based on MRI‐derived tear characteristics, which may facilitate prognosis and inform decision‐making regarding expected RTP duration in professional football players.

**Level of Evidence:**

Level IV, retrospective cohort study.

## Graphical abstract

One‐sentence summary of key findings: Clustering analysis identified seven structurally distinct hamstring injury phenogroups with various return‐to‐play durations in professional football players.

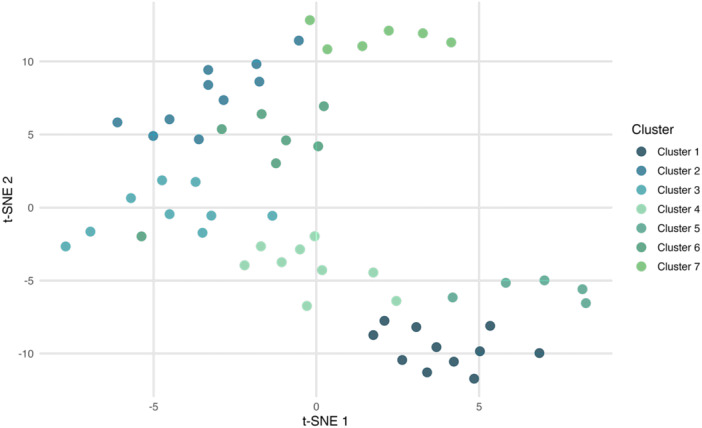

**Abbreviations**
ARI: adjusted rand index
BAMIC: British Athletics Muscle Injury Classification
CHAMP: CHecklist for statistical Assessment of Medical Papers
CSA: cross‐sectional area
Ferencvárosi TC: Ferencvárosi Torna Club
IQR: interquartile range
ML: machine learning
MRI: magnetic resonance imaging
PAM: partitioning around medoids
RTP: return to play
SD: standard deviation
STROBE: Strengthening the Reporting of Observational Studies in Epidemiology
t‐SNE: t‐distributed stochastic neighbour embedding
UEFA: Union of European Football Associations

## INTRODUCTION

The increasing prevalence of hamstring injuries in professional football imposes a considerable burden on players, organisations and healthcare staff on‐ and off‐season. Data from the European Centre for Injury Prevention shows that hamstring injuries have doubled their proportion of total injuries from 12% to 24% during a 21‐year study period, while their contribution to injury burden increased from 10% to 20% in terms of days missed due to injury [6]. The two predominant injury mechanisms for hamstring injury include sprint‐type injuries occurring during the late swing phase of running due to excessive eccentric muscle strain, and stretch‐type injuries caused by extensive hip flexion with an extended knee [4]. Sprint injuries typically affect the biceps femoris, while stretch injuries predominantly involve the semimembranosus and are associated with longer recovery times [10]. The anatomical location of injury is equally distributed between proximal (34%), central (33.4%) and distal (32.6%) sites [10].

Despite advances in imaging and classification systems, accurately predicting return to play (RTP) timeframes remains challenging. Traditional clinical assessment provides limited prognostic value, with only moderate evidence supporting associations between visual analogue scale pain scores and clinician predictions with actual RTP times [24]. While imaging‐based classification systems such as the British Athletics Muscle Injury Classification (BAMIC) [12] and MLG‐R (mechanism of injury [M], location of injury [L], grading of severity [G], and number of muscle re‐injuries [R]) [28] have shown promise in correlating injury severity with recovery duration, substantial variability exists in individual patient outcomes even within the same injury grade.

The heterogeneity in hamstring injury presentations and recovery patterns suggests that current approaches may oversimplify the complex interplay of factors influencing healing and RTP. Key determinants associated with prolonged recovery include stretching‐type injury mechanisms, structural injuries with myotendinous junction involvement, active range of motion deficits exceeding 20–25 degrees, delayed initial consultation beyond 1 week, and higher initial pain scores [9]. However, the prognostic value of individual magnetic resonance imaging (MRI) abnormalities remains inconsistent across studies, with conflicting evidence regarding the predictive utility of muscle oedema length, cross‐sectional area involvement and tendon injury characteristics [2, 12].

Machine learning (ML) methods, such as cluster analysis, represent alternative approaches to address this complexity by determining clinically meaningful subgroups of patients based on multiple demographic and injury‐specific characteristics [33]. This unsupervised ML method has demonstrated success in enhancing interpretability of patient subpopulations and improving outcome predictions across various medical conditions, making it particularly suited for precision medicine applications [5, 20, 25, 26, 31]. By grouping patients with similar injury patterns and characteristics into distinct phenogroups, cluster analysis may reveal previously unrecognised relationships between patient factors and recovery trajectories.

While the burden of overuse and trauma‐related muscle injuries is growing, there is a lack of data‐driven methods to provide athletes, healthcare professionals and coaching staff with a reliable estimate of time missed due to injury based on injury characteristics. The objective of this pilot study was to determine clinically relevant subgroups of professional football players with hamstring injuries using cluster analysis based on demographic and injury‐related characteristics, and to assess RTP duration with respect to the characteristics of these phenogroups. While the proposed study is exploratory by design, it was hypothesised that patients with higher BAMIC injury grades and more severe MRI tear characteristics would demonstrate longer recovery times and cluster into distinct phenogroups with prolonged RTP durations.

## METHODS

### Study design and ethical approval

This retrospective cohort study analysed professional football players from Ferencvárosi Torna Club (Ferencvárosi TC) between 2018 and 2024. The study adhered to the ethical principles for medical research as outlined in the Declaration of Helsinki. This study was approved by the Institutional Review Board at Uzsoki Street Hospital, Budapest, Hungary (registration number: 138‐IK/2026). Data collection was conducted in accordance with the UEFA Elite Club Injury Study (ECIS), started in 2001, and was performed by qualified physical therapists at Ferencvárosi TC. Furthermore, MRI‐derived quantitative and qualitative data were extracted by two board‐certified musculoskeletal radiologists. The study was reported in accordance with the Strengthening the Reporting of Observational Studies in Epidemiology (STROBE) guidelines for cohort studies [7].

### Participants

**Figure 1:**
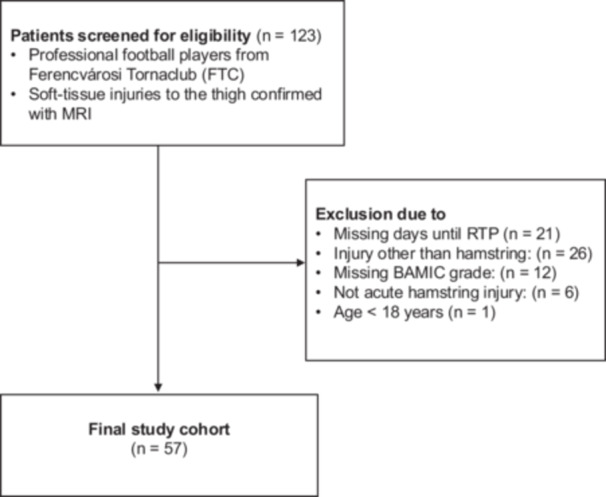
Flowchart of the inclusion and exclusion of patients with hamstring injury in the final study cohort. BAMIC, British Athletics Muscle Injury Classification; MRI, magnetic resonance imaging; RTP, return to play.

A total of 123 Hungarian Division 1 professional football players from Ferencvárosi TC with MRI‐verified soft‐tissue injuries to the thigh were screened for eligibility. Inclusion criteria were: (1) professional football players from Ferencvárosi TC, (2) soft‐tissue injuries to the thigh confirmed by MRI. Exclusion criteria included missing information regarding days until RTP without restriction (*n* = 21), injuries other than hamstring strains (*n* = 26), missing BAMIC grade (*n* = 12), non‐acute injuries (*n* = 6) and player age < 18 years (*n* = 1). The final study population comprised 57 patients with acute hamstring injuries (Figure 1).

### Imaging and clinical assessment

MRI examinations were performed using either 1.5 Tesla (*n* = 60) or 3 Tesla (*n* = 63) scanners. Radiological assessment and documentation were performed by two independent radiologists with subspecialty training in musculoskeletal imaging. All athletes underwent standardised physiotherapy at Ferencvárosi TC under the supervision of qualified sports physiotherapists.

### The British Athletics Muscle Injury Classification (BAMIC) grade

The BAMIC [21] is an evidence‐based approach to muscle injury classification based on specific MRI criteria, including cross‐sectional area of muscle oedema, craniocaudal length of muscle oedema, muscle architecture fibre disruption and tendon involvement characteristics [12]. Injury severity grading based on the BAMIC is conducted on an ordinal scale from 0: negative MRI, (1) mild muscle injury, defined as <10% cross‐sectional area (CSA) involvement and <5 cm craniocaudal length, (2) moderate muscle injury, defined as 10%–50% CSA involvement and 5–15 cm craniocaudal length, (3) severe muscle injury, defined as >50% CSA involvement and >15 cm craniocaudal length and (4) complete muscle tear [8]. Additional letter designations (a: myofascial, b: myotendinous junction, c: intratendinous) specify the extent of tissue involvement of the corresponding injury grade [8]. The BAMIC demonstrates excellent inter‐rater and intra‐rater reliability (*κ* = 0.65–1.00) [8].

### Data collection

The following variables were systematically collected into a spreadsheet from injury and radiological reports: patient characteristics (age at time of injury, days to RTP without restriction), injury characteristics (laterality, affected muscle, BAMIC grade, anatomical location, tear extensiveness, anatomical tear site) and quantitative MRI measurements (longitudinal tear length in centimeters, tear cross‐sectional area as percentage of total muscle cross‐section). In the event of uncertainty or ambiguity regarding a qualitative or quantitative parameter in the radiologist report, the extracted parameter was labelled missing.

### Statistical analysis

Data preprocessing, analyses and visualisation were performed using R (Version 4.2.3) and RStudio (Version 1.1.463). Missing data patterns were assessed using the VIM package. Missing values in longitudinal tear length (5.17%, *n* = 3) and tear cross‐sectional area (65.52%, *n* = 38) were addressed using median imputation. Statistical analyses were conducted in accordance with the CHecklist for statistical Assessment of Medical Papers (CHAMP) statement [17].

### Clustering methodology

Three clustering approaches were implemented: (1) K‐means clustering on standardised numerical variables, (2) hierarchical clustering using Ward's linkage method and (3) mixed‐type clustering using Gower distance with partitioning around medoids (PAM). The optimal number of clusters was determined using silhouette analysis, where average silhouette width reflects the magnitude of intra‐cluster homogeneity and inter‐cluster heterogeneity [15, 23]. Silhouette width ranges from −1 to 1, with values closer to 1 indicating superior internal cohesion and separation from neighbouring clusters. While there are no clearly defined cutoffs in the literature, average silhouette width values > 0.70 generally correspond to ‘strong’, 0.50–0.70 to ‘acceptable’, and 0.25–0.50 to ‘weak’ cluster separation. Negative values indicate too many or too few clusters. Clustering method performance was compared based on average silhouette width and clustering similarity was compared with the adjusted rand index (ARI), with values ranging from −1 to 1, where 1 corresponds to identical cluster partitioning, and −1 to systematically discordant cluster partitioning [13, 27]. Following a quantitative assessment of cluster separation and quality, mixed‐type clustering was selected for subsequent analysis, as it was the only clustering method capable of assessing both numerical and categorical variables for cluster separation, and may be less susceptible to outliers, which was considered superior to other methods in terms of the clinical interpretability [16, 22, 30].

### Statistical comparisons

Between‐cluster differences were assessed using Kruskal–Wallis tests for continuous variables and chi‐square tests for categorical variables. When significant overall differences were detected, post‐hoc analyses were performed using Dunn's test with Bonferroni correction for continuous variables and pairwise chi‐square tests with Bonferroni correction for categorical variables. Effect sizes were calculated using Cohen's *d*. Statistical significance was set at *p* < 0.05.

## RESULTS

### Patient demographics and injury characteristics

The study cohort comprised 57 professional football players (Table 1) with a mean age of 26.4 ± 4.8 years (range, 18–38 years). The median time to RTP was 21.0 days (interquartile range, 9.5–42.0 days). The biceps femoris was involved in 75.4% of cases (*n* = 43), followed by semitendinosus (12.3%, *n* = 7) and semimembranosus (12.3%, *n* = 7). Right‐sided injuries occurred more frequently (55.2%, *n* = 32) than left‐sided injuries (43.1%, *n* = 25). The most common BAMIC grades were 2b (25.9%, *n* = 15) and 2a (24.1%, *n* = 14). Moderate tear extensiveness was observed in 53.4% of cases (*n* = 31), while myofascial tears were most frequent (60.3%, *n* = 35).

**Table 1:** Baseline demographic and injury characteristics in the study cohort of professional football players with hamstring injuries.

Characteristic	
Demographics	
Age at injury, mean ± SD (years)	26.4 ± 4.8
Age range (years)	18–38
Injury characteristics	
Laterality, *n* (%)	
Right	31 (54.4)
Left	25 (43.9)
Bilateral	1 (1.7)
Affected muscle, *n* (%)	
Biceps femoris	43 (75.4)
Semitendinosus	7 (12.3)
Semimembranosus	7 (12.3)
BAMIC grade distribution, *n* (%)	
Grade 1a	11 (19.3)
Grade 1b	6 (10.5)
Grade 2a	13 (22.8)
Grade 2b	15 (26.3)
Grade 2c	4 (7.0)
Grade 3a	1 (1.8)
Grade 3b	2 (3.5)
Grade 3c	4 (7.0)
Grade 4	1 (1.8)
Tear extensiveness, *n* (%)	
Mild	19 (33.3)
Moderate	30 (52.6)
Extensive	4 (7.0)
Complete	4 (7.0)
Anatomical tear site, *n* (%)	
Myofascial	34 (59.6)
Myotendinous	18 (31.6)
Tendinous	5 (8.8)
Injury localisation, *n* (%)	
Proximal third	18 (31.6)
Middle third	24 (42.1)
Distal third	15 (26.3)
MRI measurements	
Lesion length, mean ± SD (cm)	8.9 ± 5.1
	*n* = 54
Cross‐sectional area involvement, median (IQR) (%)	25.0 (13.8–41.3)
	*n* = 20
Return to play, median (IQR) (days)	21.0 (9.5–42.0)

*Note*: For each variable, *n* = 57, unless otherwise specified.

Abbreviations: BAMIC, British Athletics Muscle Injury Classification; IQR, interquartile range; MRI, magnetic resonance imaging; SD, standard deviation.

### Cluster analysis

**Figure 2:**
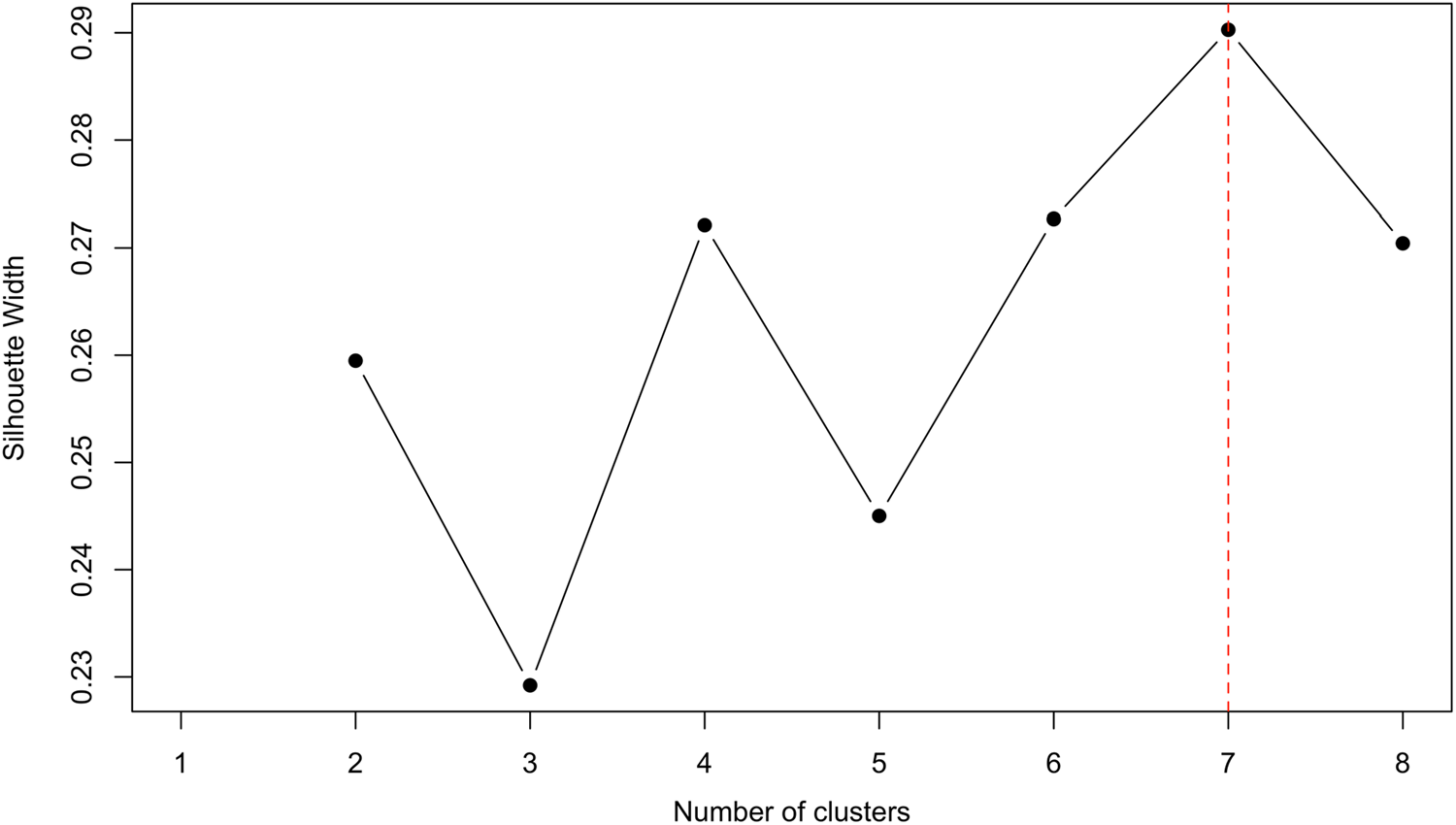
Dot plot displaying silhouette width analysis to determine the optimal number of clusters for mixed‐type clustering using partitioning around medoids.

**Figure 3:**
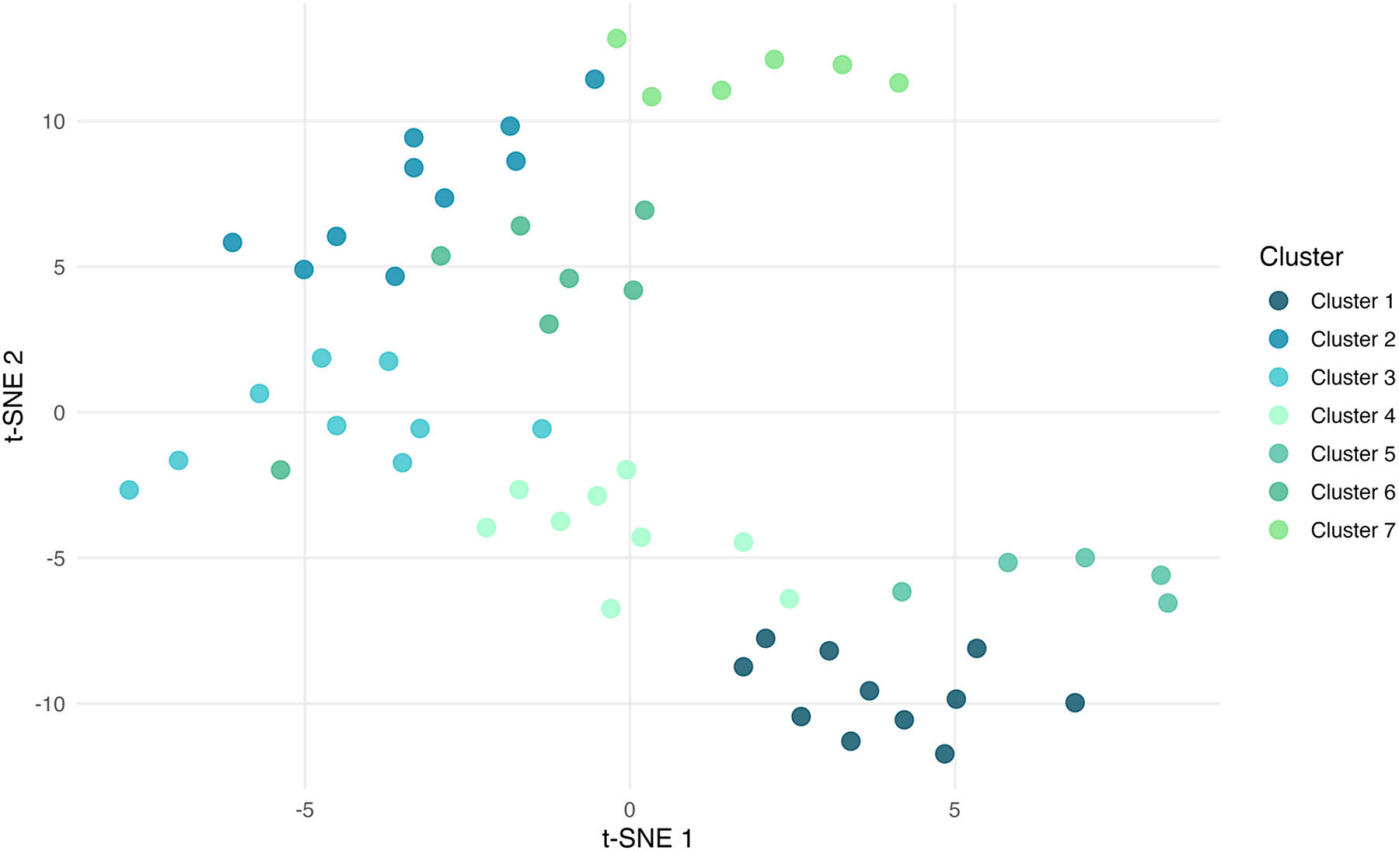
Cluster separation and overlap in mixed‐type hamstring injury data visualised using t‐distributed stochastic neighbour embedding (t‐SNE) dimensionality reduction. Each dot corresponds to a patient in the study cohort and is coloured by assigned cluster. Spatial proximity indicates similarity across all injury characteristics.

Based on the Silhouette Method for optimal *k*, the optimal number of clusters was determined to be two for *k*‐means and hierarchical clustering. The silhouette method determined seven optimal clusters for mixed‐type clustering using Gower distance with PAM (Figures 2 and 3, Table 2). Average silhouette width for hierarchical clustering was 0.370, compared with 0.342 and 0.290 for k‐means clustering and mixed‐type clustering using Gower distance with PAM, respectively (Table 2).

**Table 2:** Comparison of clustering method performance in terms of average silhouette width and cluster similarity.

Method	Number of clusters	Average silhouette width^a^	Data type	ARI^b^		
				ARI with K‐means	ARI with HC	ARI with PAM
K‐means	2	0.342	Numerical	1.000	0.447	0.069
Hierarchical (Ward)	2	0.370	Numerical	0.447	1.000	0.040
PAM (Gower)	7	0.290	Mixed (numerical and categorical)	0.069	0.040	1.000

^a^ Greater silhouette width indicates more clearly defined clusters.

^b^ ARI, adjusted rand index. Values range from −1 to 1, where 1 indicates perfect agreement between the two clusterings, 0 indicates agreement equivalent to random chance, and negative values indicate worse than random agreement.

### Cluster 1 (*n* = 11, 19.3%)

Mean patient age was 30.0 years, with a median time to RTP of 9.0 days. This cluster was characterised by mild injury extensiveness (100%) with BAMIC grade 1a (90.9%), predominantly involving the biceps femoris (90.9%), with exclusively myofascial tissue involvement, localisation to the middle third of the involved muscle in 63.6% of cases, and a mean lesion length of 4.7 cm.

### Cluster 2 (*n* = 10, 17.5%)

Mean patient age was 23.8 years, with a median time to RTP of 46.5 days. This cluster was characterised by moderate injury extensiveness (90.0%) with BAMIC grade 2b (80.0%), predominantly involving the biceps femoris (70.0%), with exclusively myotendinous tissue involvement (100%), localisation to the proximal third of the involved muscle in 60.0% of cases, and a mean lesion length of 10.9 cm.

### Cluster 3 (*n* = 9, 15.8%)

Mean patient age was 24.4 years, with a median time to RTP of 21.0 days. This cluster was characterised by moderate injury extensiveness (88.9%) with BAMIC grade 2a (66.7%), predominantly involving the biceps femoris (77.8%), with exclusively myofascial tissue involvement (100%), localisation to the middle third of the involved muscle in 88.9% of cases, and a mean lesion length of 9.8 cm.

### Cluster 4 (*n* = 9, 15.8%)

Mean patient age was 26.6 years, with a median time to RTP of 27.0 days. This cluster was characterised by moderate injury extensiveness (66.7%) with BAMIC grade 2a (77.8%), exclusively involving the biceps femoris (100%), with exclusively myofascial tissue involvement (100%), localisation to the distal third of the involved muscle in 100% of cases, and a mean lesion length of 8.6 cm.

### Cluster 5 (*n* = 5, 8.8%)

Mean patient age was 26.8 years, with a median time to RTP of 15.0 days. This cluster was characterised by mild injuries (100%) with BAMIC grade 1b (100%), predominantly involving the biceps femoris (60.0%), with predominantly myotendinous tissue involvement (80.0%), equally distributed between the distal and proximal thirds (40.0% each), and a mean lesion length of 4.6 cm.

### Cluster 6 (*n* = 7, 12.3%)

Mean patient age was 27.4 years, with a median time to RTP of 21.0 days. This cluster was characterised by moderate injuries (100%) with BAMIC grade 2b (71.4%), predominantly involving the semimembranosus (85.7%), with predominantly myofascial tissue involvement (57.1%), localisation to the proximal third of the involved muscle in 57.1% of cases, and a mean lesion length of 9.1 cm.

### Cluster 7 (*n* = 6, 10.5%)

Mean age was 25.5 years, with a median time to RTP of 94.5 days. This cluster was characterised by complete tears (66.7%) with BAMIC grade 3c (66.7%), exclusively involving the biceps femoris (100%), with predominantly tendinous tissue involvement (66.7%), localisation to the proximal third of the involved muscle in 66.7% of cases, and a mean lesion length of 15.1 cm.

### Statistical comparisons between clusters

**Figure 4:**
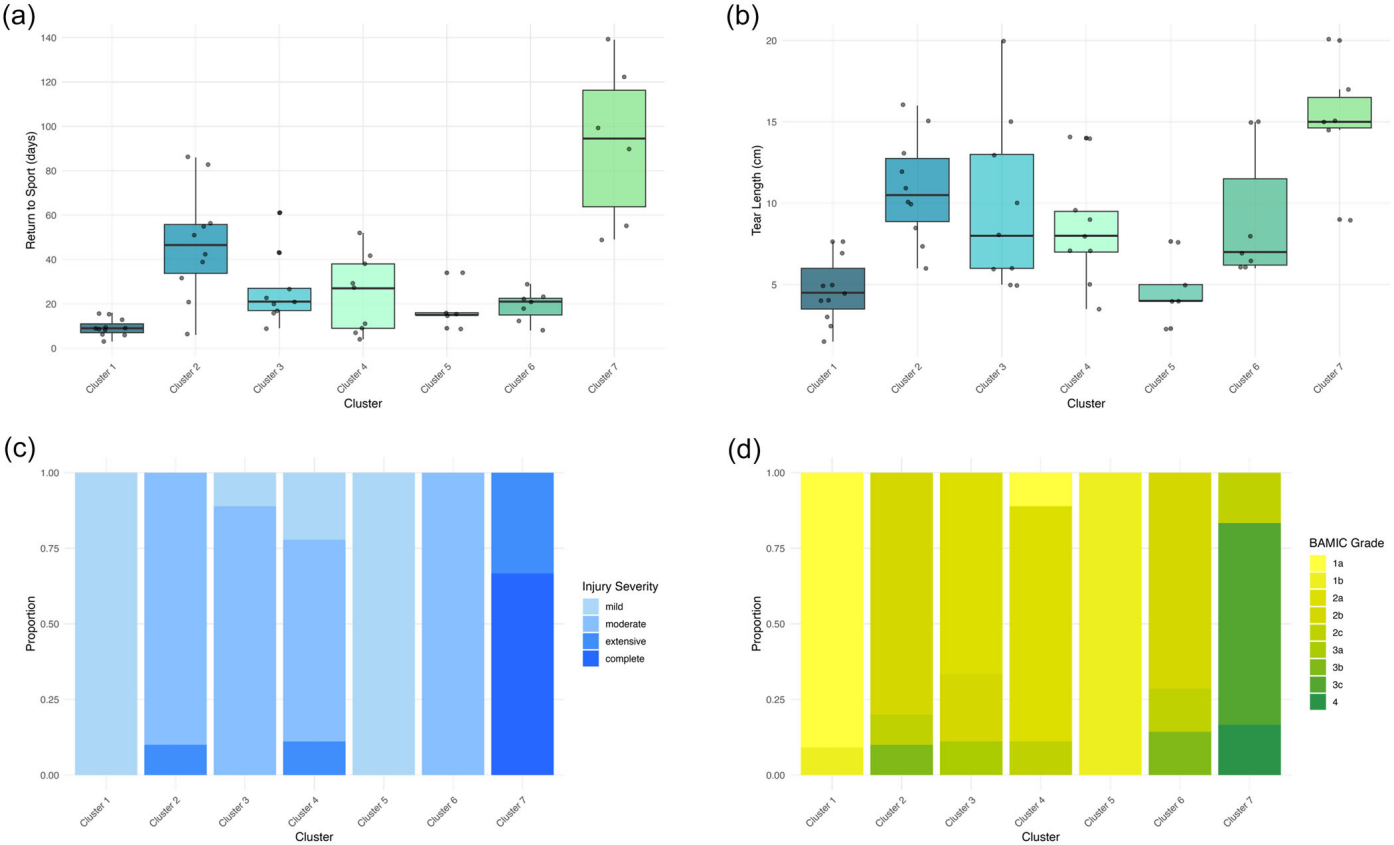
(a) Boxplot displaying the distribution of return to play duration across hamstring injury patient clusters. Boxes correspond to distributions, while points correspond to individual observations. (b) Boxplot displaying the distribution of hamstring tear length (cm) across hamstring injury patient clusters. Boxes correspond to distributions, while points correspond to individual observations. (c) Vertical bar chart displaying injury severity distribution across hamstring injury patient clusters. (d) Vertical bar chart displaying BAMIC injury grade across hamstring injury patient clusters. BAMIC, British Athletics Muscle Injury Classification.

Significant between‐cluster differences were observed for days to RTP (Figure 2), lesion length, BAMIC grade, injury severity (Figure 4) and anatomical tear site (Table 3). No significant differences were found for age, cross‐sectional area, or affected muscle.

**Table 3:** Cluster characteristics and RTP duration.

Cluster characteristic	Cluster 1	Cluster 2	Cluster 3	Cluster 4	Cluster 5	Cluster 6	Cluster 7
*n* (%)	11 (19.3)	10 (17.5)	9 (15.8)	9 (15.8)	5 (8.8)	7 (12.3)	6 (10.5)
Age, mean ± SD (years)	30.0 ± 4.1	23.8 ± 4.1	24.4 ± 5.0	26.6 ± 5.0	26.8 ± 4.3	27.4 ± 5.2	25.5 ± 4.3
RTP, median (IQR) (days)	9.0 (7.0–11.0)	46.5 (33.8–55.8)	21.0 (17.0–27.0)	27.0 (9.0–38.0)	15.0 (15.0–16.0)	21.0 (15.0–22.5)	94.5 (63.8–116.2)
RTP range (days)	3–16	6–86	9–61	4–52	9–34	8–29	49–139
BAMIC grade, *n* (%)							
1a	10 (90.9)	—	—	1 (11.1)	—	—	—
1b	1 (9.1)	—	—	—	5 (100)	—	—
2a	—	—	6 (66.7)	7 (77.8)	—	—	—
2b	—	8 (80.0)	2 (22.2)	—	—	5 (71.4)	—
2c	—	1 (10.0)	—	1 (11.1)	—	1 (14.3)	1 (16.7)
3a	—	—	1 (11.1)	—	—	—	—
3b	—	1 (10.0)	—	—	—	1 (14.3)	—
3c	—	—	—	—	—	—	4 (66.7)
4	—	—	—	—	—	—	1 (16.7)
Tear extensiveness, *n* (%)							
Mild	11 (100)	—	1 (11.1)	2 (22.2)	5 (100)	—	—
Moderate	—	9 (90.0)	8 (88.9)	6 (66.7)	—	7 (100)	—
Extensive	—	1 (10.0)	—	1 (11.1)	—	—	2 (33.3)
Complete	—	—	—	—	—	—	4 (66.7)
Anatomical tear site, *n* (%)							
Myofascial	11 (100)	—	9 (100)	9 (100)	1 (20.0)	4 (57.1)	—
Myotendinous	—	10 (100)	—	—	4 (80.0)	2 (28.6)	2 (33.3)
Tendinous	—	—	—	—	—	1 (14.3)	4 (66.7)
Affected muscle, *n* (%)							
Biceps femoris	10 (90.9)	7 (70.0)	7 (77.8)	9 (100)	3 (60.0)	1 (14.3)	6 (100)
Semitendinosus	1 (9.1)	2 (20.0)	2 (22.2)	—	2 (40.0)	—	—
Semimembranosus	—	1 (10.0)	—	—	—	6 (85.7)	—
Injury localisation, *n* (%)							
Distal third	3 (27.3)	—	—	9 (100)	2 (40.0)	1 (14.3)	—
Middle third	7 (63.6)	4 (40.0)	8 (88.9)	—	1 (20.0)	2 (28.6)	2 (33.3)
Proximal third	1 (9.1)	6 (60.0)	1 (11.1)	—	2 (40.0)	4 (57.1)	4 (66.7)
Lesion length, mean ± SD (cm)	4.7 ± 2.0	10.9 ± 3.2	9.8 ± 5.2	8.6 ± 3.6	4.6 ± 2.0	9.1 ± 4.1	15.1 ± 3.6
Cross‐sectional area, median (%)	25	25	25	25	25	25	25

*Note*: — indicates no events in the corresponding category.

Abbreviations: BAMIC, British Athletics Muscle Injury Classification; IQR, interquartile range; RTP, return to play; SD, standard deviation.

### Post‐hoc analysis

Significant pairwise differences in days to RTP were determined between Cluster 1 and Cluster 2 (*p* = 0.001, Table 4) and Cluster 1 and Cluster 7 (*p* < 0.001). Significant differences in tear length were found between Cluster 1 and Cluster 2 (*p* = 0.007), Cluster 1 and Cluster 7 (*p* < 0.001) and Cluster 5 and Cluster 7 (*p* = 0.004). Multiple significant pairwise differences in anatomical tear site were observed between Cluster 7 and several other clusters, including Cluster 1 (*p* = 0.002), Cluster 3 (*p* = 0.006) and Cluster 4 (*p* = 0.006). Significant pairwise differences in injury severity were found between Cluster 3 and Cluster 7 (*p* = 0.004) and Cluster 4 and Cluster 7 (*p* = 0.013). Significant differences in affected muscle distribution were observed between Cluster 1 and Cluster 6 (*p* = 0.008) and Cluster 3 and Cluster 6 (*p* = 0.018). Despite overall significance for BAMIC grade distribution (*p* < 0.001), pairwise comparisons could not be calculated due to sparse sample size across subgroups (Appendixes S1–S5).

**Table 4:** Between‐cluster comparison of demographic and injury‐related variables.

Variable	Test statistic	*p*‐Value
Age	*χ* ^2^ = 12.0	0.0630^a^
RTP (days)	*χ* ^2^ = 30.6	<0.001^a^
Lesion length (cm)	*χ* ^2^ = 18.4	0.0001^a^
Cross‐sectional area (%)	*χ* ^2^ = 7.3	0.295^a^
BAMIC grade	*χ* ^2^ = 188.2	<0.001^b^
Tear extensiveness	*χ* ^2^ = 93.0	<0.001^b^
Anatomical tear site	*χ* ^2^ = 73.5	<0.001^b^
Affected muscle	*χ* ^2^ ≈ 48.0	<0.001^b^

Abbreviations: BAMIC, British Athletics Muscle Injury Classification; RTP, return to play.

a The Kruskal–Wallis test was used to compare the distribution of non‐parametric continuous variables between groups.

b The chi‐square test was used to compare the distribution of categorical variables between clusters. Post‐hoc analyses were performed for variables with *p* < 0.05 using Dunn's test with Bonferroni correction for continuous variables and pairwise chi‐square tests with Bonferroni correction for categorical variables.

### Cluster validation

The overall silhouette analysis revealed an average silhouette width of 0.290, indicating weak to moderate cluster separation with some overlap between groups. Individual cluster silhouette widths ranged from 0.22 to 0.54, with Cluster 1 displaying the highest internal cohesion (0.54; Table 5). The t‐SNE visualisation confirmed partial cluster separation with overlapping boundaries, consistent with the moderate silhouette values observed. Clusters showed distinct profiles based on the comparison of standardised quantitative variables (Figure 4).

**Table 5:** Cluster validation with silhouette analysis.

Cluster	*n*	Average silhouette width
Cluster 1	11	0.51
Cluster 2	10	0.25
Cluster 3	9	0.22
Cluster 4	9	0.34
Cluster 5	5	0.28
Cluster 6	7	0.07
Cluster 7	6	0.26
Overall	57	0.290

*Note*: Silhouette width ranges from −1 to 1, with values closer to 1 indicate with superior internal cohesion and separation from neighbouring clusters.

## DISCUSSION

**Figure 5:**
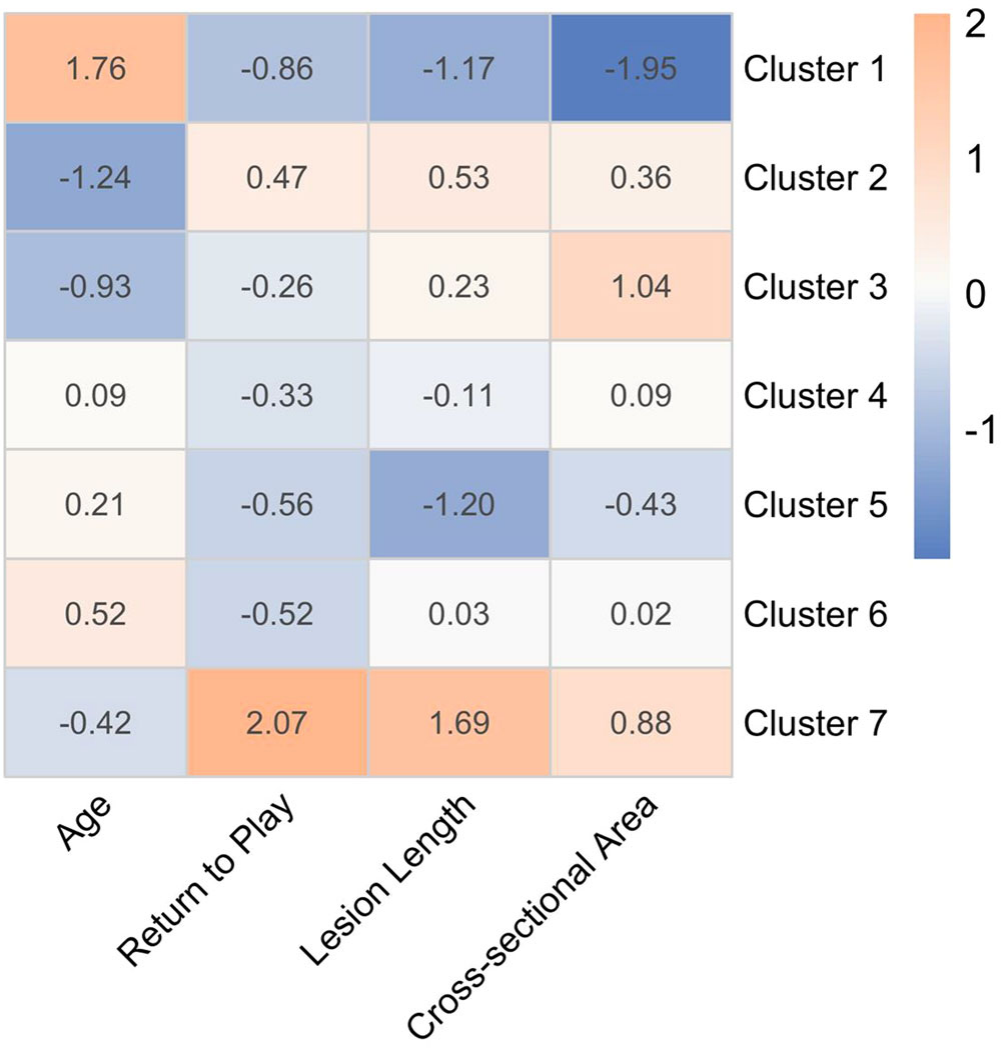
Standardised cluster profiles, with lesion length and return to play as key factors for between‐cluster differentiation. The heatmap displays standardised values (*Z*‐scores) across clusters, with colour intensity corresponding to the magnitude of deviation from the cohort mean. Shades of blue indicate below‐average values, white indicates near‐average values, and shades of orange indicate above‐average values.

The major finding of the presented study was that unsupervised clustering was able to determine seven statistically unique phenogroups of professional football players with hamstring injury based on differences in terms of demographics, radiologic variables, BAMIC injury grade and time until RTP (Figure 5). Clusters 1, 3, 4, 5 and 6 were characterised by low‐ to moderate‐grade qualitative and quantitative hamstring injury parameters and relatively quick RTP after injury (medians between 9 and 27 days). In contrast, clusters 2 and 7 displayed high‐grade qualitative and quantitative injury parameters and considerably greater delay until RTP (medians of 46.5 and 94.5 days, respectively). Between cluster differences were nuanced with respect to hamstring tear localisation, RTP duration and hamstring tear length on MRI, which may compromise clinical interpretability and actionability.

Previous univariate or multivariate analyses of clinical and radiologic parameters have been of limited prognostic value for the estimation of RTP duration after hamstring injury. A prospective cohort of 180 male athletes with hamstring injury found that MRI as an adjunct to clinical examination added only a 2.8% explanation of variance in terms of RTP duration [29]. Other factors previously associated with prolonged recovery include a stretching‐type injury mechanism, myotendinous junction involvement, and active range of motion deficits exceeding 20–25 degrees [9]. While the current study supports the prognostic significance of the extent of structural damage and connective tissue involvement on RTP duration, relevance of radiologic imaging parameters on RTP duration remains debated in the current literature [11, 14, 19, 32]. Evidence regarding the prognostic value of quantitative T2 mapping and diffusion tensor imaging remains inconclusive, and quantitative methods show limited advantage compared with conventional qualitative MRI assessment methods [2, 18]. However, craniocaudal tear length, and muscle injury site—both in terms of the involved tissue and proximodistal localisation along the longitudinal axis of the specific muscle—may be of clinical relevance, as suggested by the presented clustering analysis.

One recent study comparing supervised ML (linear regression, random forest and XGBoost) approaches to predict RTP in 76 professional football players with hamstring injuries, reported a positive association of injury grade and myotendinous junction involvement with prolonged time to RTP across all modelling approaches [28]. In contrast to regression‐based approaches, the current study determined seven distinct patient phenogroups based on combinations of potentially relevant clinical and radiologic variables, among which BAMIC grade, lesion length and anatomical tear site were distinguishing characteristics of recovery trajectory between phenogroups. Taken together, clustering may potentially capture complex, non‐linear relationships among qualitative and quantitative variables that regression models are not able to, which presents opportunities for outcome prediction based on patient and injury characteristics. Furthermore, unsupervised and supervised approaches may complement one another: unsupervised clustering may be useful in terms of exploring the underlying population structure and may identify previously unrecognised hamstring injury phenotypes, whereas supervised ML may optimise predictive accuracy for time to RTP in individual patients with hamstring injury [33].

### Clinical implications

The identification of hamstring injury phenogroups based on patient and MRI characteristics may support clinical assessment and prognostics through the data‐driven estimation of RTP timelines. Injuries involving proximal localisation, myotendinous or tendinous structures, and greater lesion length were associated with prolonged RTP. Incorporating cluster‐based stratification into clinical workflows may assist in counselling both athletes and coaching staff and may help guide individualised rehabilitation planning for professional football players with hamstring injuries.

### Strengths and limitations

The current study has several strengths and limitations. A small sample size of 57 is a major limitation, as previous simulation‐based, empirical studies report that the number of observations required for reliable effect sizes in clustering are considerably greater (at least 20–30 observations for each expected subgroup) [1, 3]. Therefore, the presented results, at this stage, may only be interpreted as a proof‐of‐concept. In general, the structure of the identified clusters was weakly to moderately defined, with relatively fuzzy boundaries (silhouette width 0.29), most likely due to sample size limitations and the complexity of a high‐dimensional dataset. Over half of the patients included in the analysis lacked information regarding tear cross‐sectional area, which makes this variable of limited reliability and relevance for the analysis. While seven unique clusters were deemed most optimal mathematically, this number may be suboptimal from the perspective of clinical interpretability and decision‐support. The inclusion of additional relevant demographic, sport‐specific and injury‐related variables, such as previous hamstring injury, time of the season, playing position, number of previous games played during the season, among others is warranted to improve the granularity of the analysed dataset and to enhance clinical relevance for RTP prediction. Future research should focus on prospective validation of phenogroups in larger, multicenter cohorts. The development of interactive clinical decision‐support tools using clustering outputs could guide prognosis and rehabilitation strategies. Finally, reinjury rate and career impact may be potential long‐term outcomes of interest in hamstring injury phenogrouping studies beyond injury characteristics and time to RTP.

## CONCLUSION

Unsupervised ML may help determine clinically relevant hamstring injury patient groups based on tear length, BAMIC grade and anatomical localisation, which may facilitate decisions regarding clinical management, and prognosis regarding the duration of time until RTP.

## AUTHOR CONTRIBUTIONS

All listed authors have contributed substantially to this work: review of the literature, data collection, statistical analysis and primary manuscript preparation were performed by Bálint Zsidai, György Gulácsi, Tibor Rudisch, Bence Márk Kovács, Pete Friar, Richard Nagy, Robert de Jonge and Gergely Pánics. Editing and final manuscript preparation were performed by Bálint Zsidai, Eric Hamrin Senorski, Kristian Samuelsson and Gergely Pánics. All authors have read the final manuscript and given final approval of the manuscript to be published. Each author consented to be accountable for all aspects of the research in ensuring that questions related to the accuracy or integrity of any part of the work are appropriately investigated and resolved.

## CONFLICT OF INTEREST STATEMENT

Kristian Samuelsson is a member on the board of directors for Getinge AB (publ) and is a medical technology advisor for Carl Bennet AB. The remaining authors declare no conflicts of interest.

## FUNDING INFORMATION

The authors have no funding to report.

## ETHICS STATEMENT

The study adhered to the ethical principles for medical research as outlined in the Declaration of Helsinki. This study was approved by the Institutional Review Board at Uzsoki Street Hospital, Budapest, Hungary (registration number: 138‐IK/2026). Data were collected within the framework of the UEFA Elite Club Injury Study (ECIS), which was approved by the UEFA Medical Committee and the UEFA Football Development Division. Patients or members of the public were not involved in the design, conduct, reporting, or dissemination plans of this research. This study included professional male football players from a single European club. While the study population reflects the target population of professional football players, it does not capture diversity across sex, competition level, or geographic regions. No specific analyses were performed to assess differences related to ethnicity or socioeconomic background. Participation in the UEFA Elite Club Injury Study (ECIS) already includes the provision of informed consent at the time of player inclusion in the surveillance program. Players are informed that their anonymized injury and exposure data may be used for research purposes within the ECIS framework. Therefore, for analyses based on ECIS registry data, it is generally accepted that informed consent has been obtained as part of the study participation.

## Supporting information

Supporting File 1.

Supporting File 2.

Supporting File 3.

## Data Availability

The data that support the findings of this study are not publicly available due to institutional and data protection restrictions but may be made available upon reasonable request and with permission from the UEFA Elite Club Injury Study.
