# Supplementary material for: Clustering analysis of hamstring injuries—A pilot study to determine phenogroups based on injury characteristics and recovery duration
Source: J Exp Orthop. 2026 Aug 17;13(3):e70885. doi: 10.1002/jeo2.70885 (PMC13479470; doi:10.1002/jeo2.70885)
Supplement: Supplementary file 1 [file JEO2-13-e70885-s002.pdf]

Appendix. CHAMP: CHecklist for statistical Assessment of Medical Papers

Design and conduct

1.	Clear description of the goal of research, study objective(s), study design, and study population	Yes	Unclear	No
2.	Clear description of outcomes, exposures/treatments and covariates, and their measurement methods	Yes	Unclear	No
3.	Validity of study design	Yes	Unclear	No
4.	Clear statement and justification of sample size	Yes	Unclear	No
5.	Clear declaration of design violations and acceptability of the design violations	Yes	Unclear	No
6.	Consistency between the paper and its previously published protocol	Yes	Unclear	No

Data analysis

7.	Correct and complete description of statistical methods	Yes	Unclear	No
8.	Valid statistical methods used and assumptions outlined	Yes	Unclear	No
9.	Appropriate assessment of treatment effect or interaction between treatment and another covariate	Yes	Unclear	No
10.	Correct use of correlation and associational statistical testing	Yes	Unclear	No
11.	Appropriate handling of continuous predictors	Yes	Unclear	No
12.	Confidence intervals do not include impossible values	Yes	Unclear	No
13.	Appropriate comparison of baseline characteristics between the study arms in randomized trials	Yes	Unclear	No
14.	Correct assessment and adjustment of confounding	Yes	Unclear	No
15.	Avoiding model extrapolation not supported by data	Yes	Unclear	No
16.	Adequate handling of missing data	Yes	Unclear	No

Reporting and presentation

17.	Adequate and correct description of the data	Yes	Unclear	No
18.	Descriptive results provided as occurrence measures with confidence intervals, and analytic results provided as association measures and confidence intervals along with P-values	Yes	Unclear	No
19.	Confidence intervals provided for the contrast between groups rather than for each group	Yes	Unclear	No
20.	Avoiding selective reporting of analyses and P-hacking	Yes	Unclear	No
21.	Appropriate and consistent numerical precisions for effect sizes, test statistics, and P-values, and reporting the P-values rather their range	Yes	Unclear	No
22.	Providing sufficient numerical results that could be included in a subsequent meta-analysis	Yes	Unclear	No
23.	Acceptable presentation of the figures and tables	Yes	Unclear	No

Interpretation

24.	Interpreting the results based on association measures and 95% confidence intervals along with P-values, and correctly interpreting large P-values as indecisive results, not evidence of absence of an effect	Yes	Unclear	No
25.	Using confidence intervals rather than post-hoc power analysis for interpreting the results of studies	Yes	Unclear	No
26.	Correctly interpreting occurrence or association measures	Yes	Unclear	No
27.	Distinguishing causation from association and correlation	Yes	Unclear	No
28.	Results of pre-specified analyses are distinguished from the results of exploratory analyses in the interpretation	Yes	Unclear	No
29.	Appropriate discussion of the study methodological limitations	Yes	Unclear	No
30.	Drawing only conclusions supported by the statistical analysis and no generalization of the results to subjects outside the target population	Yes	Unclear	No
