# Supplementary material for: Clustering analysis of hamstring injuries—A pilot study to determine phenogroups based on injury characteristics and recovery duration
Source: J Exp Orthop. 2026 Aug 17;13(3):e70885. doi: 10.1002/jeo2.70885 (PMC13479470; doi:10.1002/jeo2.70885)
Supplement: Supplementary file 2 [file JEO2-13-e70885-s001.docx]

**Appendix:**

| **Appendix 1.** Post-hoc analysis of RTP duration using Dunn's Test with Bonferroni correction between hamstring injury clusters | | | | |
| --- | --- | --- | --- | --- |
| **Cluster Comparison** | **Z-statistic** | **p-value** | **p-adjusted (Bonferroni)** | **Effect size (Cohen's d)** |
| Cluster 1 vs Cluster 2 | 4.003 | **<0.0001** | **0.0013** | −2.159 |
| Cluster 1 vs Cluster 3 | 2.614 | 0.0089 | 0.1877 | −1.532 |
| Cluster 1 vs Cluster 4 | 1.996 | 0.0460 | 0.9656 | −1.252 |
| Cluster 1 vs Cluster 5 | 1.274 | 0.2027 | 1.0000 | −1.393 |
| Cluster 1 vs Cluster 6 | 1.776 | 0.0757 | 1.0000 | −1.808 |
| Cluster 1 vs Cluster 7 | 4.894 | **<0.0001** | **<0.0001** | −3.975 |
| Cluster 2 vs Cluster 3 | −1.249 | 0.2118 | 1.0000 | 0.976 |
| Cluster 2 vs Cluster 4 | −1.854 | 0.0637 | 1.0000 | 1.045 |
| Cluster 2 vs Cluster 5 | −1.938 | 0.0526 | 1.0000 | 1.363 |
| Cluster 2 vs Cluster 6 | −1.806 | 0.0709 | 1.0000 | 1.411 |
| Cluster 2 vs Cluster 7 | 1.423 | 0.1547 | 1.0000 | −1.543 |
| Cluster 3 vs Cluster 4 | −0.590 | 0.5551 | 1.0000 | 0.120 |
| Cluster 3 vs Cluster 5 | −0.875 | 0.3817 | 1.0000 | 0.602 |
| Cluster 3 vs Cluster 6 | −0.628 | 0.5300 | 1.0000 | 0.566 |
| Cluster 3 vs Cluster 7 | 2.483 | 0.0130 | 0.2735 | −2.591 |
| Cluster 4 vs Cluster 5 | −0.376 | 0.7068 | 1.0000 | 0.429 |
| Cluster 4 vs Cluster 6 | −0.076 | 0.9394 | 1.0000 | 0.383 |
| Cluster 4 vs Cluster 7 | 3.011 | **0.0026** | 0.0547 | −2.614 |
| Cluster 5 vs Cluster 6 | 0.293 | 0.7696 | 1.0000 | −0.148 |
| Cluster 5 vs Cluster 7 | 2.967 | **0.0030** | 0.0631 | −2.724 |
| Cluster 6 vs Cluster 7 | 2.921 | **0.0035** | 0.0732 | −2.976 |
| RTP = return to play. Comparisons with p < 0.05 before and after Bonferroni-correction are bolded. Cohen's d interpretation: small, 0.2–0.5; medium, 0.5–0.8; large, ≥0.8. Absolute values are considered for effect size. | | | | |

| **Appendix 2.** Post-hoc analysis of tear length using Dunn's Test with Bonferroni correction between hamstring injury clusters | | | | |
| --- | --- | --- | --- | --- |
| **Cluster Comparison** | **Z-statistic** | **p-value** | **p-adjusted (Bonferroni)** | **Cohen's d** |
| Cluster 1 vs Cluster 2 | 3.585 | **0.0003** | **0.0071** | −2.318 |
| Cluster 1 vs Cluster 3 | 2.536 | 0.0112 | 0.2356 | −1.330 |
| Cluster 1 vs Cluster 4 | 2.185 | 0.0289 | 0.6066 | −1.353 |
| Cluster 1 vs Cluster 5 | −0.118 | 0.9060 | 1.0000 | 0.059 |
| Cluster 1 vs Cluster 6 | 2.262 | 0.0237 | 0.4975 | −1.451 |
| Cluster 1 vs Cluster 7 | 4.308 | **<0.0001** | **0.0003** | −3.883 |
| Cluster 2 vs Cluster 3 | −0.929 | 0.3531 | 1.0000 | 0.257 |
| Cluster 2 vs Cluster 4 | −1.272 | 0.2035 | 1.0000 | 0.684 |
| Cluster 2 vs Cluster 5 | −2.976 | **0.0029** | 0.0613 | 2.180 |
| Cluster 2 vs Cluster 6 | −0.959 | 0.3375 | 1.0000 | 0.506 |
| Cluster 2 vs Cluster 7 | 1.200 | 0.2300 | 1.0000 | −1.251 |
| Cluster 3 vs Cluster 4 | −0.334 | 0.7381 | 1.0000 | 0.272 |
| Cluster 3 vs Cluster 5 | −2.157 | 0.0310 | 0.6503 | 1.172 |
| Cluster 3 vs Cluster 6 | −0.091 | 0.9273 | 1.0000 | 0.150 |
| Cluster 3 vs Cluster 7 | 1.985 | 0.0471 | 0.9890 | −1.134 |
| Cluster 4 vs Cluster 5 | −1.875 | 0.0608 | 1.0000 | 1.258 |
| Cluster 4 vs Cluster 6 | 0.222 | 0.8247 | 1.0000 | −0.131 |
| Cluster 4 vs Cluster 7 | 2.285 | 0.0223 | 0.4691 | −1.811 |
| Cluster 5 vs Cluster 6 | 1.977 | 0.0481 | 1.0000 | −1.305 |
| Cluster 5 vs Cluster 7 | 3.715 | **0.0002** | **0.0043** | −3.505 |
| Cluster 6 vs Cluster 7 | 1.964 | 0.0496 | 1.0000 | −1.547 |
| Comparisons with p < 0.05 before and after Bonferroni-correction are bolded. Cohen's d interpretation: small, 0.2–0.5; medium, 0.5–0.8; large, ≥0.8. Absolute values are considered for effect size. | | | | |

| **Appendix 3.** Post-hoc analysis of anatomical tear site using pairwise Chi-square tests with Bonferroni correction between hamstring injury clusters | | | |
| --- | --- | --- | --- |
| **Cluster Comparison** | **Chi-square** | **p-value** | **p-adjusted (Bonferroni)** |
| Cluster 1 vs Cluster 2 | — | — | — |
| Cluster 1 vs Cluster 6 | 5.657 | 0.0591 | 0.5910 |
| Cluster 1 vs Cluster 7 | 17.000 | **0.0002** | **0.0020** |
| Cluster 2 vs Cluster 3 | — | — | — |
| Cluster 2 vs Cluster 4 | — | — | — |
| Cluster 2 vs Cluster 6 | 10.119 | **0.0063** | 0.0635 |
| Cluster 2 vs Cluster 7 | — | — | — |
| Cluster 3 vs Cluster 6 | 4.747 | 0.0931 | 0.9314 |
| Cluster 3 vs Cluster 7 | 15.000 | **0.0006** | **0.0055** |
| Cluster 4 vs Cluster 6 | 4.747 | 0.0931 | 0.9314 |
| Cluster 4 vs Cluster 7 | 15.000 | **0.0006** | **0.0055** |
| Cluster 5 vs Cluster 6 | 3.223 | 0.1996 | 1.0000 |
| Cluster 5 vs Cluster 7 | 5.622 | 0.0601 | 0.6014 |
| Cluster 6 vs Cluster 7 | 5.757 | 0.0562 | 0.5622 |
| Comparisons with p < 0.05 before and after Bonferroni-correction are bolded. — indicates insufficient observations for chi-square test. Pairwise comparisons test differences in distribution of myofascial, myotendinous, and tendinous injury locations between clusters. | | | |

| **Appendix 4.** Post-hoc analysis of injury severity using pairwise Chi-square tests with Bonferroni correction between hamstring injury clusters | | | |
| --- | --- | --- | --- |
| **Cluster Comparison** | **Chi-square** | **p-value** | **p-adjusted (Bonferroni)** |
| Cluster 1 vs Cluster 2 | — | — | — |
| Cluster 1 vs Cluster 3 | — | — | — |
| Cluster 1 vs Cluster 4 | — | — | — |
| Cluster 1 vs Cluster 5 | — | — | — |
| Cluster 1 vs Cluster 6 | — | — | — |
| Cluster 1 vs Cluster 7 | — | — | — |
| Cluster 2 vs Cluster 3 | — | — | — |
| Cluster 2 vs Cluster 4 | — | — | — |
| Cluster 2 vs Cluster 5 | — | — | — |
| Cluster 2 vs Cluster 6 | — | — | — |
| Cluster 2 vs Cluster 7 | — | — | — |
| Cluster 3 vs Cluster 4 | — | — | — |
| Cluster 3 vs Cluster 5 | — | — | — |
| Cluster 3 vs Cluster 6 | — | — | — |
| Cluster 3 vs Cluster 7 | 15.000 | **0.0018** | **0.0036** |
| Cluster 4 vs Cluster 5 | — | — | — |
| Cluster 4 vs Cluster 6 | — | — | — |
| Cluster 4 vs Cluster 7 | 12.222 | **0.0067** | **0.0133** |
| Cluster 5 vs Cluster 6 | — | — | — |
| Cluster 5 vs Cluster 7 | — | — | — |
| Cluster 6 vs Cluster 7 | — | — | — |
| Comparisons with p < 0.05 before and after Bonferroni-correction are bolded. — indicates insufficient observations for chi-square test. Pairwise comparisons test differences in distribution of mild, moderate, extensive, and complete tears between clusters. | | | |

| **Appendix 5.** Post-hoc analysis of affected muscle using pairwise Chi-square tests with Bonferroni correction between hamstring injury clusters | | | |
| --- | --- | --- | --- |
| **Cluster Comparison** | **Chi-square** | **p-value** | **p-adjusted (Bonferroni)** |
| Cluster 1 vs Cluster 2 | 1.819 | 0.4027 | 1.0000 |
| Cluster 1 vs Cluster 3 | — | — | — |
| Cluster 1 vs Cluster 4 | — | — | — |
| Cluster 1 vs Cluster 5 | — | — | — |
| Cluster 1 vs Cluster 6 | 14.175 | **0.0008** | **0.0075** |
| Cluster 1 vs Cluster 7 | — | — | — |
| Cluster 2 vs Cluster 3 | 0.950 | 0.6219 | 1.0000 |
| Cluster 2 vs Cluster 4 | 3.206 | 0.2013 | 1.0000 |
| Cluster 2 vs Cluster 5 | 1.050 | 0.5916 | 1.0000 |
| Cluster 2 vs Cluster 6 | 9.849 | **0.0073** | 0.0654 |
| Cluster 2 vs Cluster 7 | 2.215 | 0.3303 | 1.0000 |
| Cluster 3 vs Cluster 4 | — | — | — |
| Cluster 3 vs Cluster 5 | — | — | — |
| Cluster 3 vs Cluster 6 | 12.444 | **0.0020** | **0.0179** |
| Cluster 3 vs Cluster 7 | — | — | — |
| Cluster 4 vs Cluster 5 | — | — | — |
| Cluster 4 vs Cluster 6 | — | — | — |
| Cluster 4 vs Cluster 7 | — | — | — |
| Cluster 5 vs Cluster 6 | 8.914 | 0.0116 | 0.1044 |
| Cluster 5 vs Cluster 7 | — | — | — |
| Cluster 6 vs Cluster 7 | — | — | — |
| Comparisons with p < 0.05 before and after Bonferroni-correction are bolded. — indicates insufficient observations for chi-square test. Pairwise comparisons test differences in distribution of biceps femoris, semitendinosus, and semimembranosus injuries between clusters. | | | |
